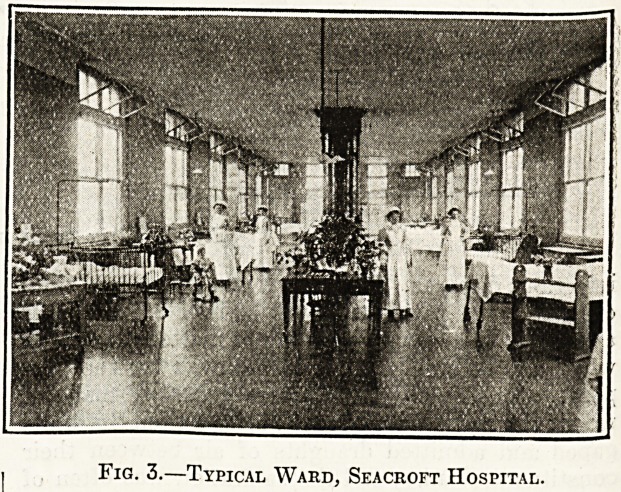# Some Fever Hospitals and Their Work

**Published:** 1912-07-20

**Authors:** A. Knyvett Gordon

**Affiliations:** formerly Medical Superintendent of Monsall Hospital and Lecturer on Infectious Diseases in the University of Manchester.


### SOME FEVER HOSPITALS AND THEIR WORK.

### II.—Preventive Methods in Provincial Institutions.

By A. Knyvett Gordon, M.B. Cantab., formerly Medical Superintendent of Monsall Hospital and Lecturer on Infectious Diseases in the University of Manchester.

In the first article of this series were outlined the principles which govern the treatment of scarlet fever in isolation hospitals; I now propose to illustrate the methods employed in a few of the larger provincial fever hospitals. I shall not attempt anything like a catalogue of these institutions, and I am well aware that there may be many where the work is excellently performed which will not be referred to. I have merely chosen some as types.

During the last fifteen years certain rather profound changes have taken place in the attitude of our municipal authorities towards the treatment of infectious diseases. Formerly it was considered sufficient that the patients should be isolated, and that as long as this was done it did not matter very much what sort of buildings were employed for the purpose. Consequently these were generally made of wood; the floors were porous; after a time the walls gaped and admitted draughts of air between their constituent planks; and the sanitation was often of the most rudimentary description. The idea in most cases, no doubt, was that they should be pulled down as soon as the epidemic during which they were hastily erected had subsided. But this excellent intention was not always carried out. The epidemic did not subside, and the wooden abominations remained.

Then a reaction took place, permanent buildings came in fashion, and some authorities erected hospitals that were structurally almost perfect, and which cost very large sums of money; they saw, in fact, that the patient had to be considered as well as the community. But the results, as far as the treatment of the patients was concerned, did not show the marked improvement that was expected (and perhaps too confidently promised to the general public); the reason, undoubtedly, was that a similarly enlightened policy had not been pursued with regard to the numbers and *personnel* of the staff. Although the idea of surgical asepsis as a goal to be reached was at the time an accepted principle in general hospitals wherever bacterial diseases were concerned, yet this was hardly sufficiently recognised in the fever hospitals, mainly because there were not enough nurses for the purpose, and partly also because sufficient attention was not always paid to the training of those who were there. It was no uncommon thing, for instance, for a hospital to accept for training young women who did not engage to stay for any definite period, and, as a matter of fact, were often merely seeking a temporary situation. They could not be expected to have that interest in the work which alone could carry them through the drudgery of an understaffed hospital.

Latterly, however, great improvements have taken place in these matters also, and I shall endeavour to show later on that the work done by the

Fig. 1.—City of Leeds Seacroft Hospital.

staff of a good provincial fever hospital is in many respects really excellent, and that extreme care is taken to safeguard the patients entrusted to its care from the risk of contracting extraneous infection.

It is necessary, however, first to refer briefly to some points in the structure of an isolation hospital, and I shall, therefore, describe the main features—in so far as they bear on the question of the securing and maintenance of surgical asepsis that is—of the

Fig. 2.—Operating Theatre at Seacroft Hospital.

Seacroft Hospital, erected in 1904 by the Corporation of Leeds (fig. 1).

This hospital is situated on a pleasant site, ninety-seven acres in extent, some distance from the heart of the city, and the rural character of the surroundings has been preserved. Nowadays, the advent of the motor-ambulance has rendered the placing of a fever hospital amongst the working quarter of a city unnecessary as well as undesirable, and in the latter situation open-air treatment of certain diseases may be almost impossible.

The hospital was designed on the pavilion system by Mr. Edwin T. Hall, and consists of forty-two separate buildings, with accommodation for 489 patients; as there was ample room on the site it was not necessary to build two-storeyed wards. The pavilions are arranged in pairs, grouped round a central administrative area, which contains the nurses' and maids' homes, kitchens, offices, etc.

The arrangement of the various buildings is very simple, and can be easily understood by reference to the accompanying plan. Complete separation of the various diseases has been secured by placing the pavilions for scarlet fever to the east of the administrative area, which divides them from the diphtheria wards on the south-west, and those for enteric fever on the west. The isolation wards are in two blocks, one to the extreme east of the scarlet fever quarter, and another south of the administrative blocks.

Each pavilion is placed axially north and south, with windows at both sides and at the ends. A road encircles the whole of the ward area, and a main corridor runs east and west throughout the hospital, on to which all the buildings are connected by glass-roofed open corridors, which give protection from the weather without risk of transference of infection from ward to ward. Under these are the subways containing pipes for steam, hot and cold water, and electric mains. Beneath each pavilion is a paved open basement about six feet high, which serves completely to disconnect the ward from the earth; in this all pipes, etc., are placed, so that repairs can be effected without entering the ward.

Each pavilion has an entrance hall, with the duty room opening from it. To the right and left are large wards each containing fourteen beds, with a floor space of 156 square feet, and a cubic air space of 2,028 cubic feet per bed. Each main ward has a single-bedded separation ward opening from it, and overlooked from the duty room or ward kitchen. There is a wide balcony at each end of the block, flanked by the sanitary towers containing the bath rooms, lavatories, and w.c.s.

The floors are of polished teak laid direct on concrete. The walls are of smooth cement, painted and varnished. The heating is by means of hot-water radiators, warmed by calorifiers in the basement, which are fed by steam from the main central boiler-house. Fresh air is passed over these at a rate calculated to change the atmosphere of the ward three times per hour. The foul air is extracted by flues heated by the fires in the ward stoves. The lighting is electric.

The surgical arrangements of the hospital are exceptionally complete. There is a separate operating block, consisting of operating theatre (fig. 2), anæsthetising and recovery rooms, which is used for major operations; and in each diphtheria pavilion there is a small ward for tracheotomies and intubations.

The aim of the architect has been to provide wards that can be rapidly and efficiently disinfected and

Fig. 3.—Typical Ward, Seacroft Hospital.

are of pleasing appearance, but without any unnecessary and expensive fads. He has certainly been remarkably successful, and it is almost impossible to find in the wards at Seacroft any corners in which dust—which in a fever hospital is simply another name for the possibility of superadded infection—can lodge, and from which it cannot be easily and quickly removed by damp sweeping or rubbing. A typical ward is illustrated here (see fig. 3).

Another important point in the administration of a large hospital is the comfort of the nursing staff, and this has been well attended to. Each nurse has a separate bedroom—a feature which all matrons will agree to be almost obligatory, but which is impossible even at the present day in many large hospitals—and there are in addition a large recreation room, a library, a writing room, and a separate sitting room for probationers. Every bedroom is heated by hot water, and the outlets for foul air from all the rooms are connected with a large shaft to which an electric fan is attached, so that " fresh air " in each bedroom is compulsory.

All the bacteriological work is done on the premises by the hospital medical staff; the laboratory for this purpose is large and thoroughly well equipped; a lecture room for students is attached.

The treatment of the sewage is another important point, and this has been exceptionally well planned. There is a separate plant for dealing with the excreta from the enteric wards, which are received into a tank and boiled by steam, after which the sewage passes into a cooling tank, and thence in a sterile condition to the main outfall sewer.

All the cooking is done by steam and gas in a large central kitchen, and in addition the bread is made on the premises in an adjoining bakery. Much cooking in the ward kitchen generally spells staff infection. The heating similarly is from a central boiler-house, and a large dynamo plant supplies all the current needed for lighting and the working of fans and any special machinery.

I have thus endeavoured to give an outline from the administrative rather than the builder's point of view of the construction of a thoroughly modern fever hospital, and I shall now proceed to deal with the clinical and administrative features of the treatment of the various infectious diseases as it is carried out in this and in some other modern hospitals, commencing with scarlet fever.

It will be noted that there are no glass cubicles for the isolation in the general wards of specially infective cases. I shall describe one of the best installations of cubicles elsewhere in a future article, and will then discuss the advantages and demerits of the system.

At the Seacroft Hospital there is ample isolation room in the separate blocks attached to the group of wards for each of the three diseases, in addition to separation wards in each pavilion. Consequently cases of co-existent scarlet fever and other infectious diseases can be treated in an isolation block, and doubtful cases—*i.e.*, where the patient is found on admission to be not certainly suffering from scarlet fever—can be accommodated in another separate block. Cases of septic scarlet fever are " barriered " in the main acute wards, each case being surrounded with a ring of wet screens, while the nurses wear overalls when inside the barrier, and either disinfect their hands before leaving it or wear rubber gloves. All utensils for the " barriered " patient are boiled, and kept separate.

One very useful feature of the Leeds City Hospitals is the Killingbeck Hospital, which is practically a miniature Seacroft as regards construction, and is situated on a separate site within easy reach of it. This building has been utilised lately for the reception of cases of scarlet fever which are found on admission to have diphtheria bacilli in the nose or throat. Every patient on admission to Seacroft is examined bacteriologically for this purpose, and though it does not follow that all of those who harbour diphtheria bacilli are suffering from diphtheria, yet they are all potential sources of infection, and the enlightened policy is of great value to the other patients.

## Figures and Tables

**Fig. 1. f1:**
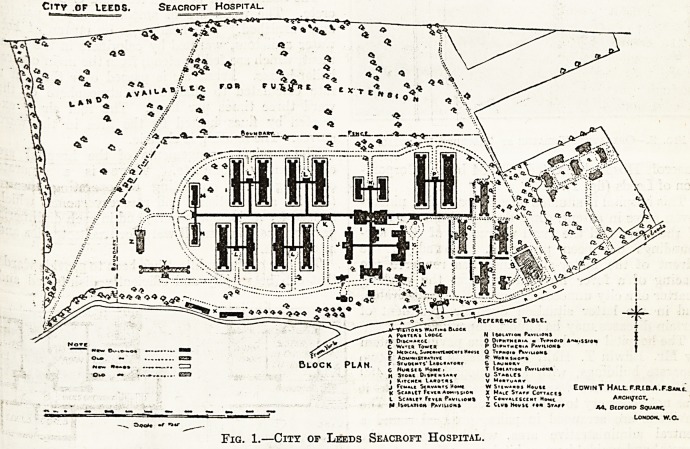


**Fig. 2. f2:**
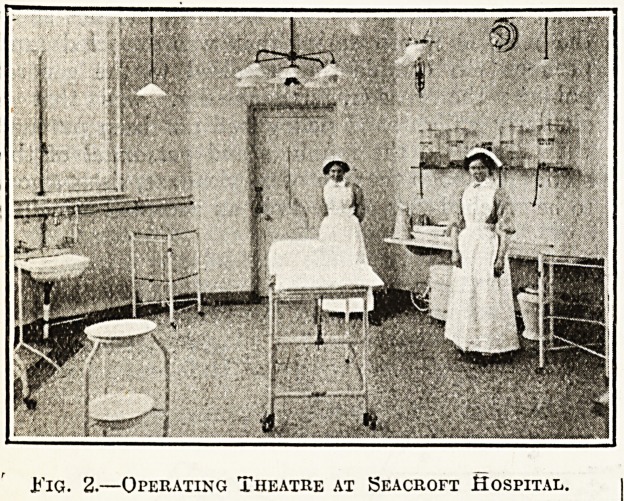


**Fig. 3. f3:**